# Depression Onset After a Spouse’s Cardiovascular Event

**DOI:** 10.1001/jamanetworkopen.2024.4602

**Published:** 2024-04-12

**Authors:** Toshiaki Komura, Yusuke Tsugawa, Naoki Kondo, Kosuke Inoue

**Affiliations:** 1 Department of Epidemiology, School of Public Health, Boston University, Boston, Massachusetts; 2 Division of General Internal Medicine and Health Services Research, David Geffen School of Medicine at UCLA, Los Angeles, California; 3 Department of Health Policy and Management, UCLA Fielding School of Public Health, Los Angeles, California; 4 Department of Social Epidemiology, Graduate School of Medicine, Kyoto University, Kyoto, Japan; 5 Hakubi Center for Advanced Research, Kyoto University, Kyoto, Japan

## Abstract

**Importance:**

Although cardiovascular disease (CVD) is a known risk factor for depression, evidence is lacking regarding whether and to what extent a spouse’s CVD is associated with the subsequent mental health of individuals.

**Objective:**

To examine the association between CVD onset in spouses and subsequent depression.

**Design, Setting, and Participants:**

This cohort study examined 277 142 matched married couples enrolled in the Japan Health Insurance Association health insurance program between April 2015 and March 2022, covering approximately 40% of the working-age population in Japan. Index individuals (primary insured) whose spouses (dependent) experienced incident CVD between April 2016 and March 2022 were 1:1 matched to controls whose spouses did not experience CVD. Matching was based on age, sex, income, or the onset date of the spouses’ CVD. Data analysis was conducted from April 2016 to March 2022.

**Exposure:**

Spousal onset of CVD between fiscal years 2016 and 2021. The *International Statistical Classification of Diseases and Related Health Problems, Tenth Revision *codes were used to identify the composite CVD outcomes (stroke, heart failure, and myocardial infarction).

**Main Outcomes and Measures:**

Multivariate Cox proportional hazards models were used to investigate the association between spouses’ new-onset CVD and individuals’ depression, adjusting for sociodemographic characteristics and comorbidities of index individuals (diabetes, hypertension, and CVD) and spouses (diabetes, hypertension, and depression). Subgroup analyses were conducted according to sex, age, income levels, and history of CVD.

**Results:**

Among 277 142 matched pairs of married couples, 263 610 (95.1%) had a male index individual; the mean (SD) age of index individuals was 58.2 (10.2) years. A new onset of depression was observed in 4876 individuals (1.8%). In multivariable Cox models, there was an association between the spouse’s CVD and the individuals’ depression (hazard ratio, 1.13 [95% CI, 1.07-1.20]). The subgroup analysis found no evidence of heterogeneity in sex, age, income level, or CVD history. The results were consistent when additionally adjusted for health behaviors (smoking, alcohol consumption, physical activity, and use of antihypertensive drugs) and objectively measured physical health conditions (body mass index, blood pressure, cholesterol levels, glucose levels, and estimated glomerular filtration rate) (hazard ratio, 1.16 [95% CI, 1.06-1.28]).

**Conclusions and Relevance:**

In this nationwide cohort study of matched couples, a spouse’s onset of CVD was associated with an increased risk of an individual’s depression. These findings highlight the importance of preventive care for mental health disorders in individuals whose spouses experience incident CVD.

## Key Points

**Question:**

Is spousal cardiovascular disease (CVD) associated with an increased risk for depression?

**Findings:**

In a nationwide cohort study with 277 142 matched pairs of married couples in Japan, spouses’ onset of CVD was associated with an increased risk of individuals’ depression. Similar patterns were found for sex, age, income, and CVD history.

**Meaning:**

These findings highlight the importance of comprehensive monitoring and preventive care for individuals after the onset of CVD among their spouses, which should be the subject of future prospective studies and clinical trials.

## Teaser

This cohort study examines the risk of depression among individuals in Japan whose spouses have been diagnosed with cardiovascular disease.

## Introduction

Mental health epidemics are a global health concern. Over the last 2 decades, the prevalence of depression surged by 64.7%, and depression ranked 13th among the top leading causes of disability-adjusted life-years in 2019, accounting for the most significant portion of mental health disorders worldwide.^1^ Alongside its negative effects on an individual’s quality of life, depression is also associated with adverse health consequences, including cardiovascular diseases (CVDs), cognitive decline, and death.^2,3^ Given the persistent increase in the number of cases,^4^ there is an urgent need to elucidate the risk factors for depression to effectively prevent it.

Although CVD is a known risk factor for depression, its relationship with depression is diverse and complex. Studies have identified various links between CVD and depression at the individual level.^5^ For example, CVD is associated with inflammation in the immune system, elevating the risk of depression.^6^ They also share common risk factors, such as obesity.^7,8^ Beyond the individual level, family members of patients with stroke experience an elevated risk of psychological symptoms, such as perceived stress, anxiety, and depression.^9,10,11^ Although these findings suggest potential associations between CVD and depression at both the individual and household levels, there are 3 major limitations in their study designs to estimate the risk. First, previous studies used opportunistic samples enrolled in a specific region or institution, which indicates potential selection bias in their results.^12,13^ Second, although evidence suggests that a longer duration of caregiving could be associated with higher chronic stress among family members,^14,15^ many studies have failed to consider the time order between occurrences of CVD and depression within households. Third, baseline adjustments for potential confounders were limited in these studies because they often recruited participants after the onset of CVD. Thus, more robust evidence is needed to comprehensively understand the relationship between CVD and depression at the household level by overcoming these limitations.

Therefore, the present study investigated the association between spouses’ onset of CVD and individuals’ depression using a large nationwide database from the Japan Health Insurance Association (JHIA). Identifying and quantifying such associations would advance our understanding of the family spillover effect of CVD on mental health and provide clinical and policy implications regarding the need for mental health management after the onset of CVD.

## Methods

This study was approved by the institutional review board of Kyoto University with a waiver of informed consent because of the retrospective nature of the data. This cohort study follows the Strengthening the Reporting of Observational Studies in Epidemiology (STROBE) reporting guideline.^16^

### Data Sources

Study samples were collected from administrative and medical records of the JHIA, the largest health insurer in Japan, which provides health care coverage to more than 30 million working-age individuals (approximately 40% of the working-age population in Japan) and their families, mainly those who work at small- to middle-sized companies and their families.^17^ The primary insured, typically the head of household, can enroll their family members as dependents to receive the same health care coverage under certain conditions, including dependents with low income (<¥1.3 million or approximately $8 700 per year). Further details are available elsewhere.^18^ The relationship between the primary insured and dependents is recorded on a self-report basis. The dataset also includes insurance claim records and annual health screening results from the beginning of fiscal year 2015 to the end of fiscal year 2021 in Japan (April 1, 2015, to March 31, 2022). All claims data were recorded monthly.

### Population

**Figure 1.**
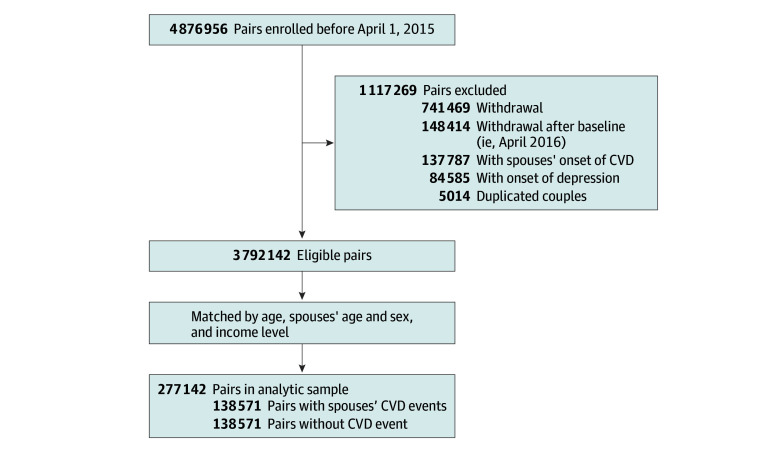
Flowchart of Study Sample Selection CVD indicates cardiovascular disease.

We enrolled index individuals who were aged 20 years and older, registered to the insurance database as the primary insured of households at the beginning of 2015, and had spouses who were registered as dependent and self-reported as husband, wife, a common-law husband, or a common-law wife. Because we used information from 2015 for baseline adjustment and started the retrospective follow-up at the beginning of 2016, we excluded index individuals who met at least 1 of the following criteria for baseline adjustment: (1) registration withdrawal before the beginning of 2016; (2) multiple spouses registered (ie, a single spouse was not identifiable); (3) a spouse received a CVD diagnosis in 2015; and (4) an index individual received a depression diagnosis in 2015. Additionally, we excluded index individuals who withdrew from registration in April 2016 (ie, those who did not complete the first month of follow-up) for postbaseline adjustment. Finally, we obtained 3 792 142 eligible pairs. Figure 1 shows the sample selection flowchart.

### Exposure

Exposure was defined as the onset of CVD, including stroke, heart failure, and myocardial infarction, in spouses between 2016 and 2021. *International Statistical Classification of Diseases and Related Health Problems, Tenth Revision *(*ICD-10*) codes recorded in the claims database were used to identify each disease based on a previous validation study (stroke: I60–63 and I64; heart failure: I50, I11.0, I13.0, and I132; and myocardial infarction: I21 and I22).^19^

### Outcome

The outcome measure was depression among index individuals between 2016 and 2021. Events were defined as diagnoses of depression using *ICD-10* codes based on a previous validation study (F32, F33.0–33.3, F33.8, F33.9, F34.1, and F41.2).^20^

### Covariates

Covariates were selected from the registration profile, insurance claims records, and annual health screenings according to a directed acyclic graph in eFigure 1 in Supplement 1. They included demographic factors (age, sex, age of spouse, and income), disease history of index individuals (diabetes, hypertension, and CVD), disease history of spouses (diabetes, hypertension, and depression), self-reported questionnaires during annual health screening (drinking status, smoking status, physical activity, and use of antihypertensive drugs), and objectively measured items during annual health screening (body mass index [BMI; calculated as weight in kilograms divided by height in meters squared], total cholesterol level, systolic blood pressure, diastolic blood pressure, high-density lipoprotein [HDL] level, serum glucose level, and estimated glomerular filtration rate [eGFR]). Details on covariate measurement can be found in eMethods 1 in Supplement 1. In analyses, each covariate was adjusted to reflect the most recent status of index individuals in the matched month during the follow-up period.

### Statistical Analysis

First, we applied matching to adjust for potential confounders and defined time zero for the unexposed group. During the follow-up period between 2016 and 2021, index individuals who were exposed were matched with those who did not receive exposure in the same month using the most recent age, sex, age of the spouse, and income. Thus, matching was conducted 72 times to include exposure incidences in the 6-year (72-month) follow-up. More details in matching are noted in eMethods 2 in Supplement 1.

Second, we estimated the associations between spouses’ onset of CVD and index individuals’ onset of depression by plotting a cumulative incidence curve. We also plotted the cumulative incidence curves for 4 subgroups: (1) matched male individuals, (2) matched female individuals, (3) matched individuals aged 20 to 59 years, and (4) matched individuals aged 60 years or older. Cumulative incidence function was adjusted with death of index individuals as a competing event.^21^

Third, multivariable Cox proportional hazards models were used to investigate the hazard ratio (HR) of depression after the onset of CVD. We adjusted multivariable Cox proportional hazards models for age; sex; age of spouse; income; index individuals’ disease histories of diabetes, hypertension, and CVD; and spouses’ disease histories of diabetes, hypertension, and depression. After performing a proportional hazard test of the model by Schoenfeld residuals, we observed a flat trend of residuals over the study period (eFigure 2 in Supplement 1). However, as the *P* value for the test was less than .05 (*P* = .02), we conducted an additional analysis using a Poisson regression model with the same set of covariates. In addition, we performed a competing risk regression analysis via Fine-Gray subdistribution hazard model to account for death of index individuals as a competing event.^22^

Fourth, to assess heterogeneity in the association, we performed subgroup analyses by sex (male and female), age (<60 years and ≥60 years), income (quartiles [Qs]), and history of CVD (yes and no). We categorized the age group as less than 60 years because Japanese citizens become eligible to receive pensions at age 60 year, which could lead to lifestyle changes.^23^ A *P* value for interaction was obtained from a product interaction term between the exposure and each characteristic (ie, sex, age, income, and history of CVD).

We also conducted additional analyses (eMethods 3 in Supplement 1). All analyses were conducted using R version 4.3.0 (R Project for Statistical Computing). Statistical significance was set at *P* < .05.

## Results

### Baseline Characteristics of the Study Population

Across the matched index individuals, the mean (SD) age was 58.15 (10.19) years, and 263 610 (95.1%) were male (Table). Although individuals in the exposed group (n = 138 571) showed a similar prevalence of a history of diseases as those in the unexposed group (n = 138 571), spouses in the exposed group were more likely to have a history of diabetes (39 869 [28.8%] vs 23 334 [16.8%]), hypertension (59 470 [42.9%] vs 34 467 [24.9%]), and depression (14 865 [10.7%] vs 8249 [6.0%]) than those in the unexposed group. Among those with available annual health screening data, objective measurements, including BMI, systolic blood pressure, diastolic blood pressure, total cholesterol level, HDL level, serum glucose level, and eGFR, showed similar distributions between the exposed and unexposed groups. Nearly one-half of index individuals were missing annual health screening data.

**Table.** Baseline Characteristics of 277 142 Individuals According to Spouse’s Cardiovascular Event

Characteristics	Index individuals, No. (%)	
	Spouse’s CVD event (n = 138 571)	No spouse’s CVD event (n = 138 571)
Age, mean (SD), y	58.15 (10.19)	58.15 (10.19)
Age of spouse, mean (SD), y	56.81 (10.35)	56.81 (10.35)
Sex		
Male	131 805 (95.1)	131 805 (95.1)
Female	6766 (4.9)	6766 (4.9)
Income quartile		
First	55 131 (39.8)	55 131 (39.8)
Second	28 604 (20.6)	28 604 (20.6)
Third	26 131 (18.9)	26 131 (18.9)
Fourth	28 705 (20.7)	28 705 (20.7)
History of diabetes	36 804 (26.6)	33 905 (24.5)
History of hypertension	53 558 (38.7)	51 497 (37.2)
History of CVD	16 836 (12.1)	13 369 (9.6)
Spouse’s history of diabetes	39 869 (28.8)	23 334 (16.8)
Spouse’s history of hypertension	59 470 (42.9)	34 467 (24.9)
Spouse’s history of depression	14 865 (10.7)	8249 (6.0)
Drinking status		
Hardly or never	19 451 (14.0)	19 123 (13.8)
Sometimes	17 608 (12.7)	17 653 (12.7)
Every day	26 990 (19.5)	27 443 (19.8)
Missing	74 522 (53.8)	74 352 (53.7)
Smoking status		
Nonsmoker	46 485 (33.5)	47 088 (34.0)
Current smoker	25 615 (18.5)	25 268 (18.2)
Missing	66 471 (48.0)	66 215 (47.8)
Physically activity		
Physically inactive	48 507 (35.0)	48 170 (34.8)
Physically active	15 080 (10.9)	15 609 (11.3)
Missing	74 984 (54.1)	74 792 (54.0)
Use of antihypertensive drug		
No	52 495 (37.9)	53 447 (38.6)
Yes	19 605 (14.1)	18 909 (13.6)
Missing	66 471 (48.0)	66 215 (47.8)
BMI, mean (SD)	23.96 (3.40)	23.84 (3.36)
Missing	66 485 (48.0)	66 222 (47.8)
Systolic blood pressure, mean (SD), mm Hg	128.13 (17.12)	128.10 (17.19)
Missing	69 107 (49.9)	68 889 (49.7)
Diastolic blood pressure, mean (SD), mm Hg	79.15 (11.44)	79.22 (11.53)
Missing	69 108 (49.9)	68 888 (49.7)
Total cholesterol, mean (SD), mg/dL	205.43 (34.10)	206.24 (34.13)
Missing	66 500 (48.0)	66 267 (47.8)
HDL, mean (SD), mg/dL	59.12 (15.80)	59.33 (15.80)
Missing	66 486 (48.0)	66 252 (47.8)
Serum glucose, mean (SD), mg/dL	103.40 (22.04)	102.96 (21.55)
Missing	74 651 (53.9)	74 292 (53.6)
eGFR, mean (SD), mL/min/1.73 m^2^	74.37 (14.30)	74.19 (14.17)
Missing	66 534 (48.0)	66 296 (47.8)

Abbreviations: BMI, body mass index (calculated as weight in kilograms divided by height in meters squared); CVD, cardiovascular disease; eGFR, estimated glomerular filtration rate; HDL, high-density lipoprotein.

SI conversion factors: To convert cholesterol and HDL to millimoles per liter, multiply by 0.0259; glucose to millimoles per liter, multiply by 0.0555.

### Spouses’ Onset of CVD and Individuals’ Depression

**Figure 2.**
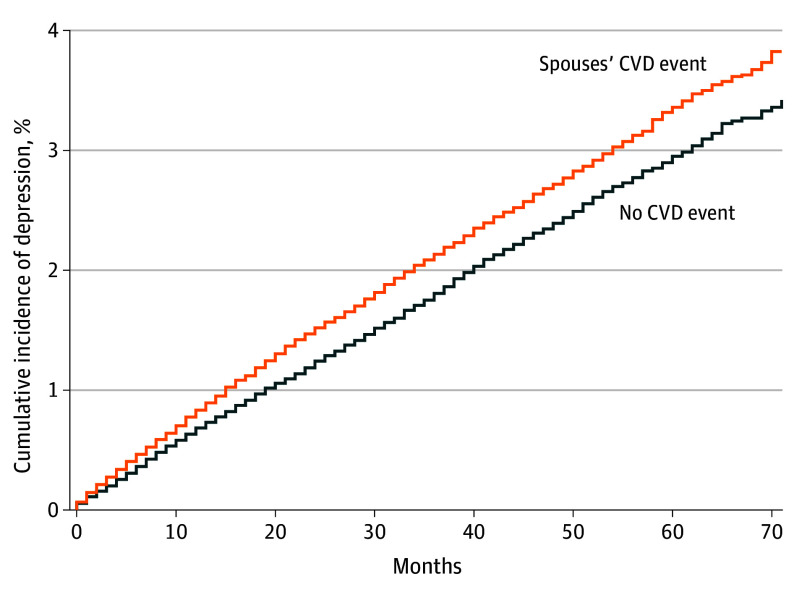
Cumulative Incidence Curve of Index Individuals’ Depression by Spouses’ Cardiovascular (CVD) Event Among the Entire Study Sample Cumulative incidence function was applied to account for death of the index individuals.

**Figure 3.**
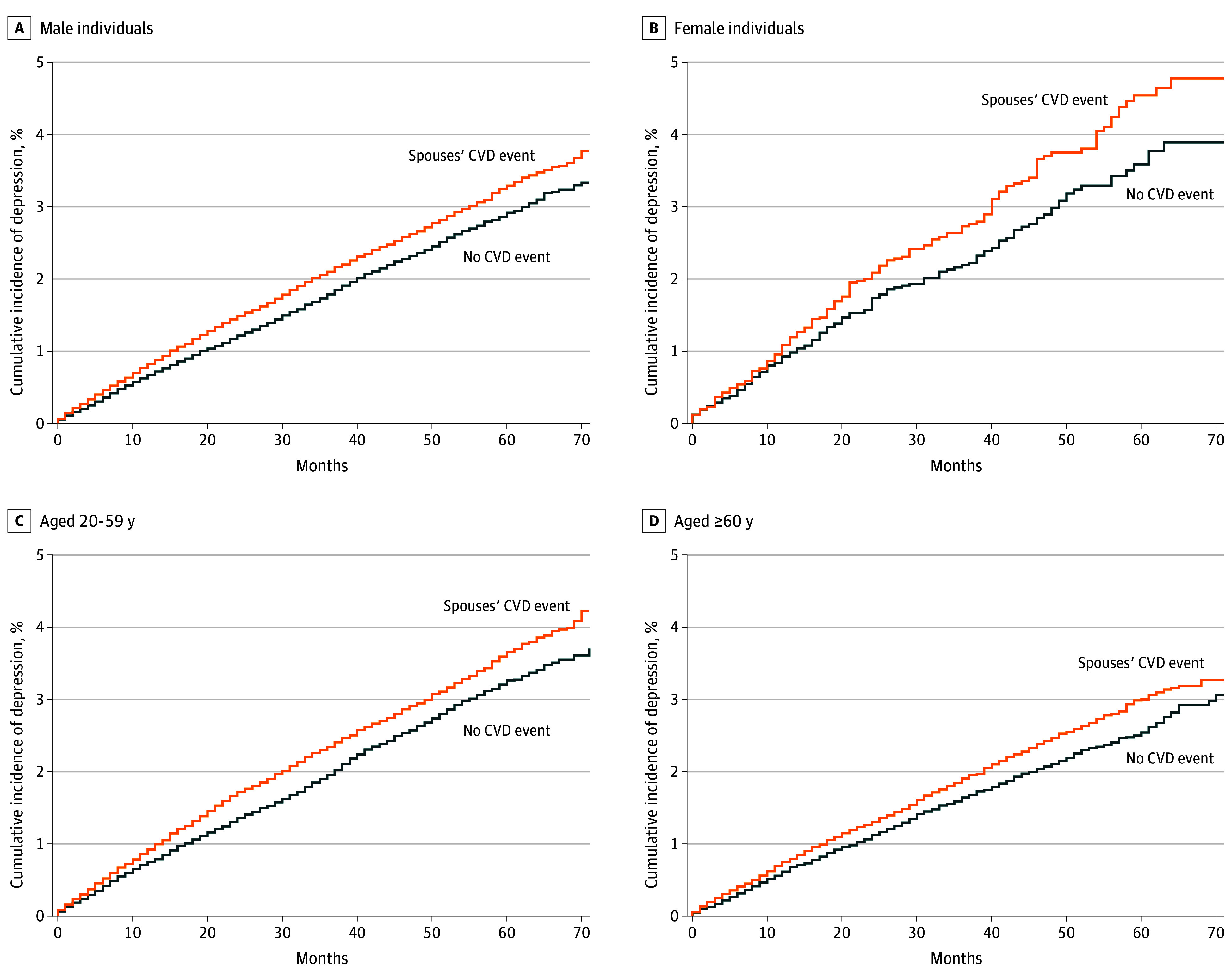
Cumulative Incidence Curves of Index Individuals’ Depression by Spouses’ Cardiovascular (CVD) Event According to Sex and Age Each panel showed cumulative incidence curves for matched male individuals (A), matched female individuals (B), matched individuals aged 20 to 59 years (C), and matched individuals aged 60 years or older (D). Cumulative incidence function was applied to account for death of the index individuals.

Over the median (IQR) follow-up period of 30 (13-49) months, there were 4876 depression diagnosis events. The cumulative incidence curves showed the trend of index individuals’ depression after the spouses’ onset of CVD (exposed) or not (unexposed) (cumulative incidence rate over the study period for exposed group, 3.8%; for unexposed group, 3.4%) (Figure 2). Overall, during the follow-up period, the exposed group had a higher cumulative incidence of depression than the unexposed group. This pattern was consistently observed among males, females, individuals aged 20 to 59 years, and those aged 60 years or older (cumulative incidence rate over the study period for exposed males, 3.8%; exposed females, 4.8%; exposed age 20-59 years, 4.2%; exposed age ≥60 years, 3.3%) (Figure 3).

**Figure 4.**
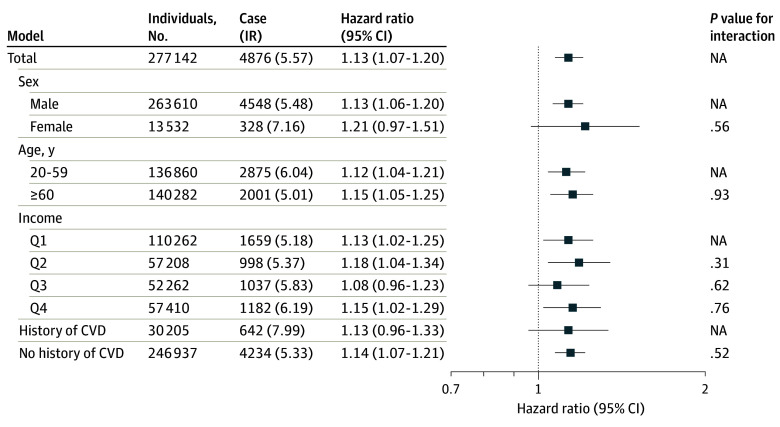
Subgroup Analysis for the Associations Between Spouses’ Cardiovascular (CVD) Event and the Individuals’ Depression The models included age; age of spouse; sex; income level; index individuals’ history of diabetes, hypertension, and CVD; and spouses’ history of diabetes, hypertension, and depression. IR indicates incidence rate; NA, not applicable; and Q, quartile.

The Cox proportional hazard analysis showed that exposed individuals had an increased risk of depression compared with the unexposed after adjusting for age; sex; age of spouse; income; index individuals’ disease histories of diabetes, hypertension, and CVD; and spouses’ disease histories of diabetes, hypertension, and depression (HR, 1.13 [95% CI, 1.07-1.20]) (Figure 4). The additional analysis using Poisson regression model showed almost the same results (incidence rate ratio, 1.13 [95% CI, 1.07-1.20]) (eFigure 3 in Supplement 1). A similar result was obtained from the Fine-Gray model (HR, 1.14 [95% CI, 1.07-1.20]). We found no evidence of heterogeneity when stratified by sex, age, income levels, and history of CVD (male: HR, 1.13 [95% CI, 1.06-1.20]; aged 20-59 years: HR, 1.12 [95% CI, 1.04-1.21]; aged ≥60 years: HR, 1.15 [95% CI, 1.05-1.25]; Q1 income: HR, 1.13 [95% CI, 1.02-1.25]; Q2 income: HR, 1.18 [95% CI, 1.04-1.34]; Q4 income: HR, 1.15 [95% CI, 1.02-1.29]; and without history of CVD: HR, 1.14 [95% CI, 1.07 to 1.21]).

### Additional Analyses

After additionally adjusting the Cox models with annual health screening data, associations between spouses’ onset of CVD and index individuals’ depression were present among all matched samples (HR, 1.16 [95% CI, 1.06-1.28]) (eFigure 4 in Supplement 1). The subgroup analysis showed no evidence of heterogeneity by sex, age, income levels, or history of CVD, while index individuals with the highest income group showed a null association between the spouses’ onset of CVD and index individuals’ depression (HR, 0.99 [95% CI, 0.82-1.19]).

When we stratified individuals by hospitalization status of the spouse, we found the associations between the spouses’ onset of CVD and individuals’ depression in both hospitalized and nonhospitalized cases (hospitalized: 71 932 cases; HR, 1.23 [95% CI, 1.10-1.38]; nonhospitalized: 205 210 cases; HR, 1.10 [95% CI, 1.03-1.18]; *P* for interaction = .12).

Lastly, on defining the exposed group for each CVD component, we found an increased risk of depression after the spouses’ onset of stroke (104 494 cases; HR, 1.13 [95% CI, 1.03-1.24]) and heart failure (201 364; HR, 1.15 [95% CI, 1.07-1.23]) (eFigure 5 in Supplement 1). There was no association between individual’s depression and spouse’s myocardial infarction (14 368 cases; HR, 0.95 [95% CI, 0.73-1.24]).

## Discussion

Using a nationwide cohort in Japan over a median follow-up period of 30 months, we found the association between spouses’ onset of CVD and increased risk of index individuals’ depression. This association was observed even after adjusting for self-reported health behaviors and objective measurements from annual health screening data, in addition to age, sex, income, disease history, and spouses’ demographic characteristics. Similar patterns were observed across strata for sex, age, income level, and history of CVD. Additionally, a modestly higher risk for depression was observed among index individuals whose spouses were hospitalized when CVD event occurred compared with those without hospitalization, although the difference was not statistically significant. Because we examined the associations between CVD and depression among couples, our findings are likely to reflect associations with psychological, behavioral, and socioeconomic factors but exclude the influence of genetics, which is also an interpersonal predictor of depression and CVD.^24^

While many public health studies have reported numerous aspects of the association between CVD and depression at the individual level,^5^ our findings add to the evidence for these associations at the household level. Particularly, a few existing studies investigating the associations between CVD and depression at the household level suffered from selection bias, unmeasured confounding, and reverse causality due to limited data.^12,13^ Specifically, to our knowledge, no studies adjusted the analysis by disease histories, health behaviors, and physical health conditions, which left a concern about unmeasured confounding. In this context, the present study fills the knowledge gap and provides more robust evidence by (1) enrolling samples from an administrative database with more than 40 million individuals, (2) matching individuals through a follow-up period of up to 6 years, and (3) adjusting for various demographic and health-related factors. The large sample size of our study also allowed us to conduct a subgroup analysis based on age, sex, income, and history of CVD.

Previous literature has highlighted that family members of patients with CVD experience numerous elements that lead to depression. First, research has reported the informal caregiving burden of patients with CVD as a risk factor for depression.^9,10^ Since informal caregiving burden causes family members to experience a lifestyle change, isolation from social life, and increased unhealthy behaviors, such as sleep disturbance and less frequent physical activity,^25,26,27,28^ the duration and intensity of caregiving are positively associated with chronic psychological stress.^14,15,29^ Moreover, family members may have to leave their work for informal home management of patients with CVD, creating financial difficulties within the household.^30,31^ Relatedly, previous research estimated the financial cost due to informal caregiving to be $616 billion in the United States as of 2015.^32^ The other pathway between the spouse’s onset of CVD and the individual’s depression is grief due to the loss of a loved one with CVD.^33^ Family members of a patient with CVD may also experience psychological distress (postintensive care syndrome) after the use of intensive care units.^34^ Lastly, studies have indicated that the stigma associated with CVD could be a potential risk factor for depression at the household level, although more research is needed in this field.^35,36^

Researchers have implemented various intervention strategies to reduce the psychological distress of family members of patients with CVD. The major types of interventions for caregiving burden include psychoeducation for patients and family members to build knowledge and skills to cope with CVD care and counseling sessions to mitigate their psychological stress.^37^ Although multiple intervention designs have been conducted, the effectiveness of intervention strategies has been inconsistent, and the most effective strategy for caregiving burden is inconclusive to date.^37,38,39^ However, previous studies identified that caregiving burdens could be modified by cultural elements such as religious activities,^40^ which implies that interpersonal pathways from the spouse’s onset of CVD to individuals’ depression are complex and contextual. Thus, desirable support in clinical practice might require a flexible framework to provide tailored support for the family members of patients with CVD. One possible form is collaborative monitoring by cardiologists and psychiatrists.^41^ Although the collaborative care model was originally designed to support the comorbidities of individual patients, such a clinical approach could also be effective at the household level. Research has shown the beneficial effects of community-level strategies to mitigate the perceived stress of caregivers of patients with Alzheimer disease, including community intervention to provide multiple supports and the implementation of virtual groups for caregivers.^42,43^ Similar structural supports could be effective for family members of individuals with CVD. Further studies are needed to understand the subpopulations with high vulnerability and the associated mechanisms to design effective frameworks that ensure the health security of family members and the prognosis of patients with CVD.

The present study has the following 3 strengths. To examine the hypothesis, we used a large administrative database that reduced the potential bias associated with opportunistic sampling and provided detailed profiles of individuals, including age, sex, income, disease history, and comprehensive measurements of physical health conditions from annual health screening data. Second, in our study design, we applied matching between the exposed and unexposed groups to control for potential confounding factors and uniquely defined the start date of follow-up based on the exposure event between 2016 and 2021, which enabled simultaneous comparisons of events that occurred on different dates. Third, we examined the associations between spouses’ onset of CVD and individuals’ depression for up to 6 years, which is, to our knowledge, the longest follow-up period among all published studies to assess these associations.

### Limitations

Our study has several limitations. First, our findings suffer from residual confounding due to the lack of information on household socioeconomic status and lifestyle behaviors of index individuals and their spouses. Second, because the JHIA database included claims and medical records since 2015, it is possible that our study did not correctly identify spouses’ new onset of CVD and individuals’ depression. However, we believe that a 1-year screening period is adequate to exclude most active cases of spouses’ CVD diagnosis and individuals’ depression. Third, our assessment of exposure, outcome, and covariates based on the associated *ICD-10* codes might induce information bias due to misclassification of these variables. However, the extent of such misclassification should be limited because the incorporated definitions of exposure and outcome were reported to have considerable positive predictive values in previous validation studies.^19,20,44,45^ Future studies addressing outcomes which often occur together with depression (eg, anxiety, change in BMI, and health behaviors) might reinforce our findings. Fourth, our analysis did not incorporate details of the spouse’s onset of CVD, including consequential mortality status, which should be the subject of future studies. Fifth, while we did not conduct the imputation of annual health screening data, the limited availability might have induced selection bias in our results. Sixth, we enrolled individuals whose spouses were registered as dependents, indicating the low-income status of their spouses in Japan. This selection of household type may limit the generalizability of our analysis to individuals with spouses who are not registered as dependents (ie, spouses who are not low income).

## Conclusions

In this cohort study of matched couples, we found associations between the spouse’s onset of CVD and an increased risk of depression among nearly 280 000 couples in Japan. Associations were present across multiple subgroups after adjusting for various individual characteristics, including age, sex, income, disease history, and comprehensive measurements of physical health conditions from annual health screening. These results indicate the need for comprehensive preventive care for individuals’ mental health after their spouses’ onset of CVD, such as cross-field collaboration between cardiologists and psychiatrists and the expansion of supportive frameworks at the community level. Further investigations are needed to address the mechanisms of the association between CVD and depression at the household level and to establish effective monitoring frameworks.

## Notes

Supplement 1. **eMethods 1.** Details in Covariate Measurement **eMethods 2.** Details in Matching **eMethods 3.** Additional Analyses **eFigure 1.** Directed Acyclic Graph Constructed for This Study **eFigure 2.** Plot of Schoenfeld Residuals to Test Proportional Hazard Assumption **eFigure 3.** Additional Analysis for the Associations Between Spouses’ Cardiovascular Event and the Individuals’ Depression Using Poisson Regression Model **eFigure 4.** Sensitivity Analysis for the Associations Between Spouses’ Cardiovascular Event and the Individuals’ Depression, Additionally Adjusting for Detailed Information Obtained From Annual Health Screening **eFigure 5.** Cumulative Incidence Curves of Index Individuals’ Depression by Spouses’ Onset of Stroke, Heart Failure, and Myocardial Infarction

Supplement 2.
Data Sharing Statement
