# Supplementary material for: Depression Onset After a Spouse’s Cardiovascular Event
Source: JAMA Netw Open. 2024 Apr 12;7(4):e244602. doi: 10.1001/jamanetworkopen.2024.4602 (PMC11015352; doi:10.1001/jamanetworkopen.2024.4602)
Supplement: Supplement 1. — eMethods 1. Details in Covariate Measurement eMethods 2. Details in Matching eMethods 3. Additional Analyses eFigure 1. Directed Acyclic Graph Constructed for This Study eFigure 2. Plot of Schoenfeld Residuals to Test Proportional Hazard Assumption eFigure 3. Additional Analysis for the Associations Between Spouses’ Cardiovascular Event and the Individuals’ Depression Using Poisson Regression Model eFigure 4. Sensitivity Analysis for the Associations Between Spouses’ Cardiovascular Event and the Individuals’ Depression, Additionally Adjusting for Detailed Information Obtained From Annual Health Screening eFigure 5. Cumulative Incidence Curves of Index Individuals’ Depression by Spouses’ Onset of Stroke, Heart Failure, and Myocardial Infarction [file jamanetwopen-e244602-s001.pdf]

Supplemental Online Content

Komura T, Tsugawa Y, Kondo N, Inoue K. Depression onset after a spouse's cardiovascular event. *JAMA Netw Open.* 2024;7(4):e244602. doi:10.1001/jamanetworkopen.2024.4602

eMethods 1. Details in Covariate Measurement

eMethods 2. Details in Matching

eMethods 3. Additional Analyses

eFigure 1. Directed Acyclic Graph Constructed for This Study

eFigure 2. Plot of Schoenfeld Residuals to Test Proportional Hazard Assumption

eFigure 3. Additional Analysis for the Associations Between Spouses' Cardiovascular Event and the Individuals' Depression Using Poisson Regression Model

eFigure 4. Sensitivity Analysis for the Associations Between Spouses' Cardiovascular Event and the Individuals' Depression, Additionally Adjusting for Detailed Information Obtained From Annual Health Screening

eFigure 5. Cumulative Incidence Curves of Index Individuals' Depression by Spouses' Onset of Stroke, Heart Failure, and Myocardial Infarction

This supplemental material has been provided by the authors to give readers additional information about their work.

eMethods 1. Details in Covariate Measurement

Income data was obtained from insurance premium profile, which recorded annual income data 50 categories, with level 1 indicating \$5,353 (696,000 yen) and level 50 indicating \$128,308 (16,680,000 yen). Income was categorized into quartiles based on comparisons among eligible index individuals.

Disease histories were obtained from claim records. Disease history, including diabetes, hypertension, CVD, and depression, was identified if index individuals had insurance claims with at least one of the ICD-10 codes from previous validation studies before exposure (diabetes: E10, E110–117, E119, E12, E130–137, E139, E14, E15, E741, E748, E831, E891, O240, O241, O244, O249, O98, P700–702, R73, and R8; hypertension: I10–13 and I15).[1,2]

Index individuals' health behaviors were determined based on their responses to questionnaires during the annual health screening. First, the drinking status was categorized into three groups: daily, sometimes, and hardly or never. Second, smoking status was determined by whether individuals currently smoked (i.e., smoked at least 100 cigarettes in total or had a smoking habit longer than six months and smoked last month). Third, individuals were classified as physically active if they had an exercise habit that met the following criteria: 1) at least moderately intense; 2) each session was longer than 30 min; 3) occurred twice a week or more; and 4) continued the habit for more than one year. Finally, we classified whether individuals used antihypertensive drugs prescribed by a doctor.

Other objectively measured annual health screening data were derived directly from the database. To calculate eGFR, we used the following formula, modified for Japanese adults: $\text{eGFR} = 194 \times \text{serum creatinine}^{-1.094} \times \text{age}^{-0.287} \times \alpha$ ($\alpha = 0.739$ for females, and $\alpha = 1$ for males).[3] We did not conduct imputation of annual health screening data because around 50% of the analytic sample was missing the information.

1. Nishioka Y, Takeshita S, Kubo S, et al. Appropriate definition of diabetes using an administrative database: A cross-sectional cohort validation study. *J Diabetes Investig*. 2022;13(2):249-255.

© 2024 Komura T et al. *JAMA Network Open.*

2. Quan H, Khan N, Hemmelgarn BR, et al. Validation of a case definition to define hypertension using administrative data. *Hypertension*. 2009;54(6):1423-1428.

3. Matsuo S, Imai E, Horio M, et al. Revised equations for estimated GFR from serum creatinine in Japan. *Am J Kidney Dis*. 2009;53(6):982-992.

eMethods 2. Details in Matching

During the follow-up period, index individuals were excluded from the matching candidates after they met at least one of the following criteria: 1) registration withdrawal of index individuals; 2) registration withdrawal of spouses (i.e., exposure status became unidentifiable); 3) spouses diagnosed with CVD (i.e., exposure occurred); and 4) index individuals who received a diagnosis of depression (outcome occurred). All the matches were performed without replacement.

© 2024 Komura T et al. *JAMA Network Open.*

eMethods 3. Additional Analyses

As a sensitivity analysis, we additionally adjusted the Cox proportional hazard models with annual health-screening data measured in the year prior to the matched month for each matched pair to minimize residual confounding bias.

Second, because mental health burden may differ by the severity of spouse's CVD event, we conducted a subgroup analysis by whether the spouse was hospitalized when CVD event occurred.

Lastly, to further investigate the differences in the associations by exposure type, we estimated the associations between spouses' onset of CVD components and index individuals' depression, respectively. After redefining the exposure status as 1) stroke, 2) heart failure, or 3) myocardial infarction, we additionally conducted the matching process three times to create three matched cohorts, in which the exposed status represented the spouses' onset of each CVD component. In this analysis, index individuals free from a specific CVD type exposure were eligible for matching as unexposed.

© 2024 Komura T et al. *JAMA Network Open.*

eFigure 1. Directed Acyclic Graph Constructed for This Study[a]

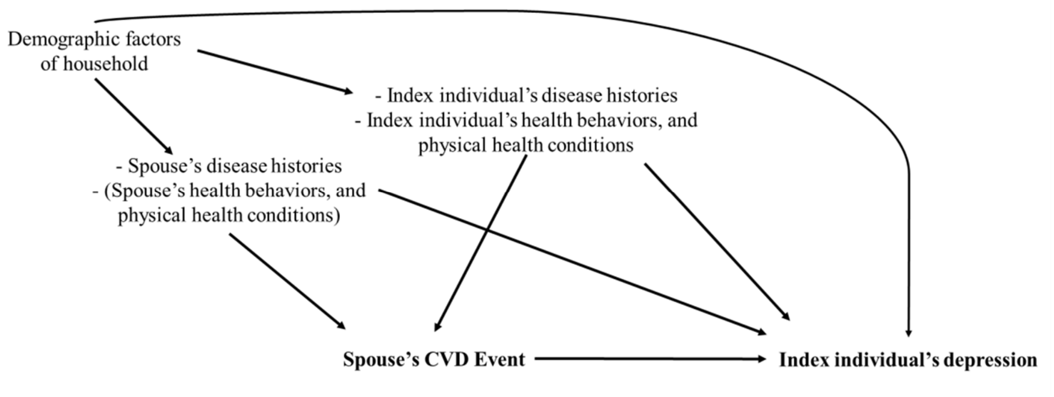

Abbreviations: CVD, cardiovascular disease.

[a] (Spouse's health behaviors, and physical health conditions) are unmeasured variables.

© 2024 Komura T et al. *JAMA Network Open.*

eFigure 2. Plot of Schoenfeld Residuals to Test Proportional Hazard Assumption[a]

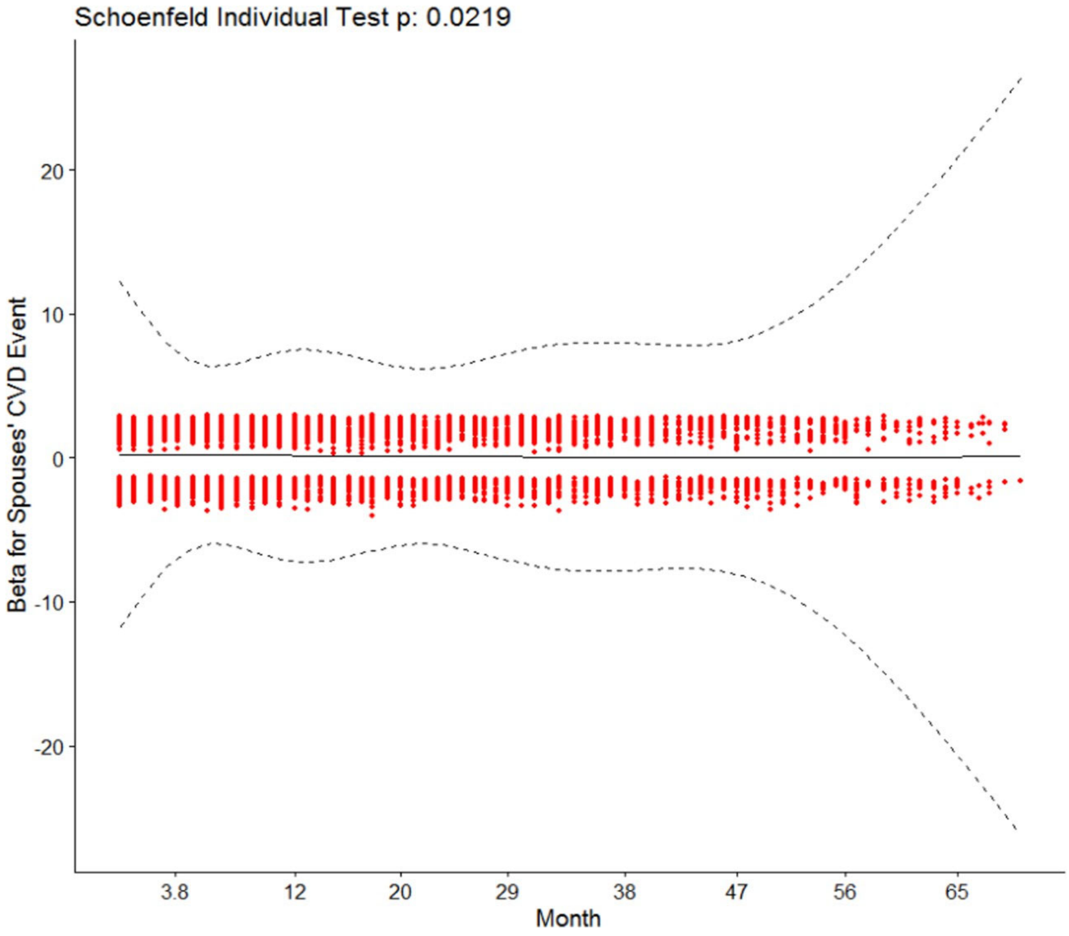

Abbreviations: CVD, cardiovascular disease.

[a] The Schoenfeld test was performed using a model included age, age of spouse, sex, income level, index individuals' history of diabetes, hypertension, and CVD, and spouses' history of diabetes, hypertension, and depression.

© 2024 Komura T et al. *JAMA Network Open.*

eFigure 3. Additional Analysis for the Associations Between Spouses' Cardiovascular Event and the Individuals' Depression Using Poisson Regression Model[a]

Model	N	IRR [95% CI]	P-value for Interaction	Case (IR)
All Sample	277,142	1.13 [1.07-1.20]	-	4,876 (5.57)
Male	263,610	1.13 [1.06-1.20]	Ref	4,548 (5.48)
Female	13,532	1.22 [0.97-1.53]	0.50	328 (7.16)
Age 20-59	136,860	1.12 [1.04-1.21]	Ref	2,875 (6.04)
Age ≥60	140,282	1.15 [1.05-1.26]	0.84	2,001 (5.01)
Income: Q1	110,262	1.12 [1.01-1.24]	Ref	1,659 (5.18)
Income: Q2	57,208	1.20 [1.06-1.37]	0.18	998 (5.37)
Income: Q3	52,262	1.07 [0.94-1.21]	0.55	1,037 (5.83)
Income: Q4	57,410	1.15 [1.02-1.30]	0.60	1,182 (6.19)
History of CVD	30,205	1.11 [0.94-1.30]	Ref	642 (7.99)
No History of CVD	246,937	1.14 [1.07-1.21]	0.36	4,234 (5.33)

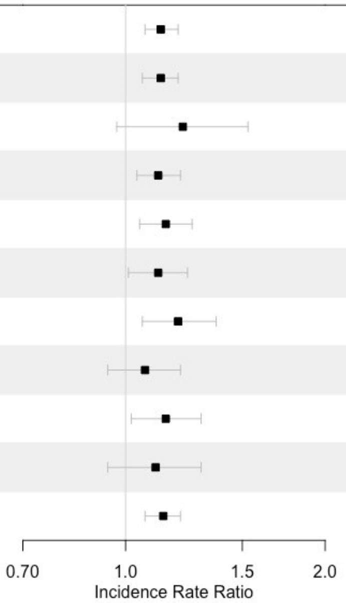

Abbreviations: IRR, incidence rate ratio; CI, confidence interval; Q, quartile; IR, incidence rate (per 10,000 person-year); and CVD, cardiovascular disease.

[a] The models included age, age of spouse, sex, income level, index individuals' history of diabetes, hypertension, and CVD, and spouses' history of diabetes, hypertension, and depression.

© 2024 Komura T et al. *JAMA Network Open.*

eFigure 4. Sensitivity Analysis for the Associations Between Spouses' Cardiovascular Event and the Individuals' Depression, Additionally Adjusting for Detailed Information Obtained From Annual Health Screening[a]

Model	N	HR [95% CI]	P-value for Interaction	Case (IR)
All Sample	108,505	1.16 [1.06-1.28]	-	1,802 (5.41)
Male	103,686	1.14 [1.03-1.26]	Ref	1,688 (5.32)
Female	4,819	1.51 [1.02-2.24]	0.30	114 (7.21)
Age 20-59	58,638	1.13 [1.00-1.27]	Ref	1,204 (6.19)
Age ≥60	49,867	1.21 [1.02-1.42]	0.65	598 (4.33)
Income: Q1	36,429	1.13 [0.94-1.35]	Ref	493 (4.81)
Income: Q2	22,902	1.42 [1.15-1.76]	0.05	382 (5.36)
Income: Q3	23,823	1.19 [0.98-1.45]	0.60	439 (5.67)
Income: Q4	25,351	0.99 [0.82-1.19]	0.37	488 (5.97)
History of CVD	10,323	1.05 [0.78-1.42]	Ref	190 (7.03)
No History of CVD	98,182	1.17 [1.06-1.30]	0.35	1,612 (5.27)

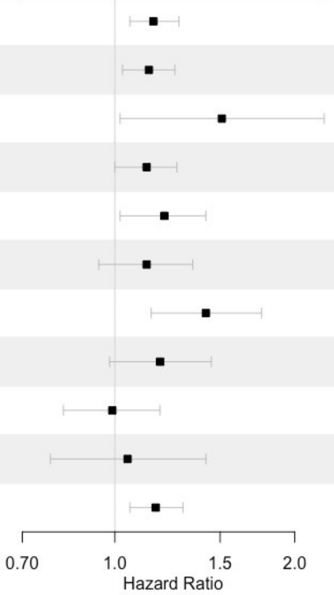

Abbreviations: HR, hazard ratio; CI, confidence interval; Q, quartile; IR, incidence rate (per 10,000 person-year); and CVD, cardiovascular disease.

[a] The models included age, age of spouse, sex, income level, index individuals' history of diabetes, hypertension, and CVD, and spouses' history of diabetes, hypertension, depression, drinking status, smoking status, physical activity, use of antihypertensive drug, BMI, systolic blood pressure, diastolic blood pressure, total cholesterol, HDL, serum glucose, and eGFR.

© 2024 Komura T et al. *JAMA Network Open.*

eFigure 5. Cumulative Incidence Curves of Index Individuals' Depression by Spouses' Onset of (A) Stroke, (B) Heart Failure, and (C) Myocardial Infarction[a]

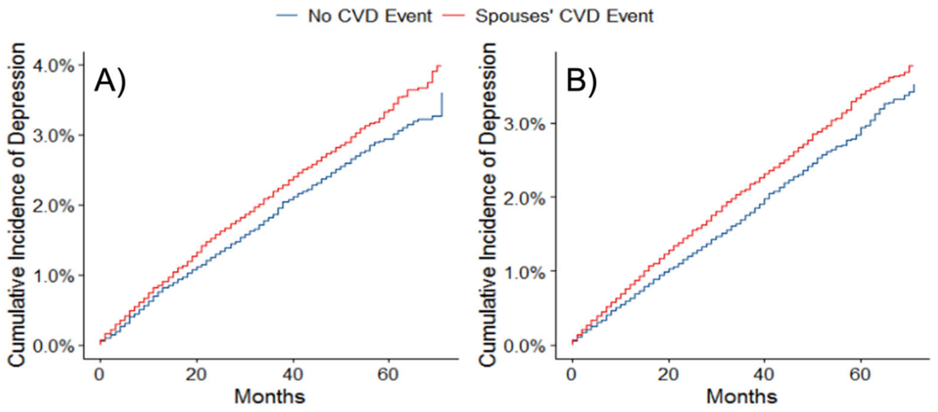

(Number at risk)	t =0	t =20	t =40	t =60
No CVD Event	104,476	67,940	38,486	13,305
CVD Event	104,524	68,280	38,864	13,808

	t =0	t =20	t =40	t =60
	201,555	127,183	68,826	23,138
	201,359	128,382	70,631	24,164

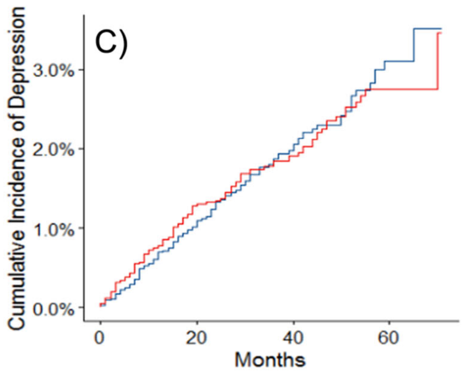

(Number at risk)	t =0	t =20	t =40	t =60
No CVD Event	14,343	9,119	5,043	1,630
CVD Event	14,388	9,221	5,172	1,738

Abbreviations: CVD, cardiovascular disease.

[a] Cumulative incidence function was applied to account for death of the index individuals.

© 2024 Komura T et al. *JAMA Network Open.*
