# Supplementary material for: Depression Onset After a Spouse’s Cardiovascular Event
Source: JAMA Netw Open. 2024 Apr 12;7(4):e244602. doi: 10.1001/jamanetworkopen.2024.4602 (PMC11015352; doi:10.1001/jamanetworkopen.2024.4602)
Supplement: Supplement 2. — Data Sharing Statement [file jamanetwopen-e244602-s002.pdf]

Data Sharing Statement

Komura. Depression Onset After a Spouse's Cardiovascular Event. *JAMA Netw Open*. Published April 12, 2024. doi:10.1001/jamanetworkopen.2024.4602

Data
Data available: No

Additional Information
Explanation for why data not available: The data that support the findings of this study are available from the Japan Health Insurance Association; however, we used these under license for the current study, and so the data are not publicly available.
